# Deuterated water (^2^H_2_O, heavy water) labelling to investigate human cell dynamics *in vivo* - lessons in protocol design and toxicity from the current literature

**DOI:** 10.3389/fimmu.2025.1544193

**Published:** 2025-04-28

**Authors:** Ami Song, Yan Zhang, Robert Busch, Becca Asquith, Derek Macallan

**Affiliations:** ^1^ Institute for Infection and Immunity, City St George's, University of London, London, United Kingdom; ^2^ School of Life and Health Sciences, Whitelands College, University of Roehampton, London, United Kingdom; ^3^ Dept of Infectious Disease, Imperial College London, London, United Kingdom; ^4^ Infection Clinical Academic Group, St George’s University Hospitals NHS Foundation Trust, London, United Kingdom

**Keywords:** humans, deuterated water, deuterium, deuterium oxide, heavy water, cell proliferation

## Abstract

The use of deuterated water (also known as ‘heavy water’) as a tracer to measure human *in vivo* cell proliferation rates for specific cell subsets has expanded significantly in recent years. Although there have been several published methods papers, investigators developing new applications may be confused by differences in study design and deuterated water dose/duration. Furthermore, this approach may be met with regulatory difficulties and participant concerns about toxicity. This scoping review explores lessons that can be learnt from the current literature on the use of deuterated water in human *in vivo* studies measuring cell proliferation. We identified 29 such studies involving 535 study participants, both healthy volunteers and those with specific clinical conditions. Wide variations in protocols were noted with doses ranging from 40-100 ml/day of pure deuterated water (or equivalent) and durations from 4-12 weeks. Study design usually reflected the kinetics of the cell of interest. No clinical toxicity signals were noted in any studies although four studies did report transient dizziness, a recognized symptom of changing water density. These published studies provide a strong safety signal for potential participants and regulatory authorities and can act as templates for the development of new research applications.

## 1 Introduction

Healthy human physiology depends upon the effective regulation of cell proliferation, survival and death. Being able to measure the rates of these processes *in vivo* safely, without interfering with the processes themselves, is foundational for understanding both physiology and pathology. Prior to the introduction of stable (non-radioactive) isotope tracer methodologies (1), techniques to measure cell turnover *in vivo*, such as the use of bromodeoxyuridine (BrdU) and ^3^H-thymidine (^3^HdT) (tracers incorporated into newly synthesized DNA during proliferation), and carboxyfluorescein succinimidyl ester (CFSE) for tracking cell division by fluorescent dye dilution, were limited for human studies by significant toxicities; they also likely perturbed the processes they were intended to measure (2). The development of a stable isotope approach to measure cell proliferation *in vivo* (1) marked a step-change in the attainability of human *in vivo* cell turnover data. This approach took well-developed deuterium tracer methods from the world of human metabolic and nutrition research and applied them to quantify DNA synthesis, the marker of cell division (or strictly cell-cycle S-phase), to derive whole cell proliferation rates. Initial studies used deuterium-labelled glucose (3), but subsequently most studies have used deuterated water (“heavy water”, deuterium oxide, ^2^H_2_O or D_2_O) as a tracer (4). Whilst deuterated water is finding new applications such as quantitative metabolomics (5), this review restricts itself to the use of deuterated water in human tracer studies for the study of *in vivo* cell kinetics.

Although there have been several excellent methodological papers (4, 6), a plethora of different approaches have been used in different centers with wide variations in study design and deuterated water dose and duration; this diversity can be confusing for investigators seeking to follow a ‘typical’ published protocol. Some variance is to be expected as protocols are tailored to the target cell of interest. For example, protocols designed to capture labelling in slow turnover cells will employ longer/higher labelling periods to achieve measurable ‘enrichments’ when compared to studies of rapidly-dividing cells, which can be captured with short labelling phases. [In this context, ‘enrichment’ has a specific meaning, being the level of isotope content above background (7)] All studies are constrained by the fact that the body water pool is very large and has a relatively slow turnover – typically 5~10% per day (8, 9). This has two implications. Firstly, it means that plateau labelling takes a long time to achieve. This time can be shortened by administering additional doses early in the labelling protocol – so called ‘priming’. Secondly, it means that deuterium persists in body water (the precursor in labelling terms) for several weeks after dosing with deuterated water stops – hence incorporation of deuterium into the product (DNA in this instance) also continues for several weeks after discontinuation of administration.

Furthermore, despite their widespread use, proposed deuterated water studies to study human cell dynamics *in vivo* may encounter regulatory difficulties and participant concerns. Some reviewers and participants are mistakenly disquieted by the radiation risk associated with an “isotope” - here it is important to recognize that “stable” isotopes are non-radioactive by definition. Furthermore, the historical association of deuterated water with nuclear armaments and nuclear energy generation, where deuterated water has been used as a fast neutron speed moderator, can also alarm potential participants – as will any internet search. Toxicity is a genuine concern as deuterium can have deleterious effects but these are only seen at very high levels, far in excess of the tracer doses used in human studies where deuterium safety has been clearly established (10, 11). Undesirable but expected physiological effects should be differentiated from toxicity. The human inner ear is so exquisitely sensitive that it can detect small changes in endolymph density which may be perceived as mild vestibular disturbance (12). Such “dizziness” arises exclusively during the initial stages of deuterated water labelling and is more likely if large doses of deuterated water are administered rapidly. Individuals vary in their sensitivity, but symptoms are transient and usually mild (11).

There is great potential to expand the scope of human *in vivo* cell dynamic studies using heavy water as they can be applied to any cell-type that can be adequately sampled. Investigators considering such studies may be unsure about applicability, be confused about which protocol to follow, and encounter concerns about safety and toxicity. In order to address these uncertainties, we set out to document the current status of published human deuterated water studies intended to measure cell proliferation *in vivo*. Our aims were to: (i) review the current range of applications of human *in vivo* deuterated water studies for cell turnover measurement; (ii) review the dose/durations/protocols used; and (iii) to collate information on possible toxicities and adverse effects. This review offers general insights into the application of the deuterated water labelling approach for future human *in vivo* cell dynamic studies and collates evidence which regulators, investigators and participants will find useful to guide their engagement in proposed studies.

## 2 Search strategy

We assessed the current use of deuterated water in human *in vivo* studies to measure cell proliferation by performing a scoping review (13) of literature published up to April 2024 in PubMed by key words, ‘deuterium oxide’, ‘heavy water’, or ‘stable isotope labeling’ and an identified key-researcher in this area (Borghans J, Hellerstein M, Macallan D, Tesselaar K), yielding 126 papers. We checked for omissions using citation lists. After excluding duplicates, 83 studies were screened. Our primary inclusion criterion was studies using the deuterated water labeling method *in vivo* in humans to measure cell kinetics. We excluded studies using deuterated water for other purposes such as body composition or energy expenditure measurement, studies using deuterium-labelled glucose only, animal studies, studies where the target was non-cellular and studies in which the number of subjects was not mentioned.

## 3 Results

We identified 29 studies which met our inclusion criteria, involving a total of 535 reported participants (**Table 1**). Some duplicates (participants featuring in more than one study) were identified; although there is some uncertainty, we estimate that at least 520 individual subjects were labeled. We analyzed these studies in terms of (i) the cells targeted; (ii) the patient groups/disease states included; (iii) the dose and duration of labelling; (iv) the modelling/normalization used; and (v) reported adverse events.

**Table 1:** Summary of publications and participants in deuterated water cell turnover studies.

ID	Authors (Year)	Title	Participants	Number in study
1	Neese et al. (2002)	Measurement *in vivo* of proliferation rates of slow turnover cells by 2H2O labeling of the deoxyribose moiety of DNA (14)	Healthy volunteers	21
2	Hellerstein et al. (2003)	Subpopulations of long-lived and short-lived T cells in advanced HIV-1 infection (15)	People ± HIV-1	30
3	Strawford et al. (2004)	Adipose tissue triglyceride turnover, *de novo* lipogenesis, and cell proliferation in humans measured with 2H2O (16)	Healthy volunteers	19
4	Messmer BT et al.*b*(2005)	*In vivo* measurements document the dynamic cellular kinetics of chronic lymphocytic leukemia B cells (17)	B-CLL patients	19
5	Misell LM et al. (2005)	Development of a novel method for measuring *in vivo* breast epithelial cell proliferation in humans (18)	Women with breast cancer and healthy volunteers	26
6	Lindwall G et al. (2006)	Heavy water labeling of keratin as a non-invasive biomarker of skin turnover *in vivo* in rodents and humans (19)	Healthy volunteers	4
7	Vrisekoop N et al. (2008)	Sparse production but preferential incorporation of recently produced naïve T cells in the human peripheral pool (20)	Healthy volunteers	5
8	Calissano C et al. (2009)	*In vivo* intraclonal and interclonal kinetic heterogeneity in B-cell chronic lymphocytic leukemia (21)	B-CLL patients	13
9	Pillay J et al. (2010)	*In vivo* labeling with 2H2O reveals a human neutrophil lifespan of 5.4 days (22)	Healthy male volunteers	5
10	Hayes GM et al. (2010)	Isolation of malignant B cells from patients with chronic lymphocytic leukemia (CLL) for analysis of cell proliferation: validation of a simplified method suitable for multi-center clinical studies (23)	B-CLL patients	29
11	Calissano C et al. (2011)	Intraclonal complexity in chronic lymphocytic leukemia: fractions enriched in recently born/divided and older/quiescent cells (24)	B-CLL patients	15
12	Hayes GM et al. (2012)	Regional cell proliferation in microdissected human prostate specimens after heavy water labeling *in vivo*: correlation with prostate epithelial cells isolated from seminal fluid (25)	Prostate cancer patients and healthy volunteers	24
13	Bollyky JB et al. (2013)	Evaluation of *in vivo* T cell kinetics: use of heavy isotope labelling in type 1 diabetes (26)	People with type 1 diabetes and healthy volunteers	20
14	Westera L et al. (2015)	Lymphocyte maintenance during healthy aging requires no substantial alterations in cellular turnover (27)	Young and elderly people	15
15	Allister CA et al. (2015)	*In vivo* 2H2O administration reveals impaired triglyceride storage in adipose tissue of insulin-resistant humans (28)	Insulin sensitive and resistant people	15
16	White UA et al. (2016)	Differences in *In Vivo* Cellular Kinetics in Abdominal and Femoral Subcutaneous Adipose Tissue in Women (29)	Women with overweight/obesity	25
17	Ahmed R et al. (2016)	Human Stem Cell-like Memory T Cells Are Maintained in a State of Dynamic Flux (30)	Healthy volunteers	7
18	Lahoz-Beneytez J et al. (2016)	Human neutrophil kinetics: modeling of stable isotope labeling data supports short blood neutrophil half-lives (31)	Healthy volunteers	4
19	Akondy RS et al. (2017)	Origin and differentiation of human memory CD8 T cells after vaccination (32)	Vaccinated individuals	37
20	Burger JA et al. (2017)	Leukemia cell proliferation and death in chronic lymphocytic leukemia patients on therapy with the BTK inhibitor ibrutinib (33)	B-CLL patients	30
21	White UA et al. (2018)	Racial differences in *in vivo* adipose lipid kinetics in humans (34)	Women with overweight/obesity	52
22	Costa Del Amo P et al. (2018)	Human TSCM cell dynamics *in vivo* are compatible with long-lived immunological memory and stemness (35)	Healthy volunteers	4
23	Ladell K et al. (2018)	Central Memory CD8+ T Cells Appear to Have a Shorter Lifespan and Reduced Abundance as a Function of HIV Disease Progression (36)	People ± HIV-1	9
24	Nouws J et al. (2019)	Altered *In Vivo* Lipid Fluxes and Cell Dynamics in Subcutaneous Adipose Tissues Are Associated With the Unfavorable Pattern of Fat Distribution in Obese Adolescent Girls (37)	Obese adolescent girls	15
25	Ahmed R et al. (2020)	CD57+ Memory T Cells Proliferate *In Vivo* (38)	People ± HIV-1	12
26	Baliu-Piqué M et al. (2021)	Cell-density independent increased lymphocyte production and loss rates post-autologous HSCT (39)	Hematologic malignancy patients	6
27	Sara P. H et al. (2021)	Quantification of T-cell dynamics during latent cytomegalovirus infection in humans (40)	CMV +/-people	10
28	White U et al. (2021)	Adipose depot-specific effects of 16 weeks of pioglitazone on *in vivo* adipogenesis in women with obesity: a randomized controlled trial (41)	Women with obesity	41
29	Zhang Y et al. (2023)	KIR-HLA interactions extend human CD8+ T cell lifespan *in vivo* (42)	People ± HIV-1, HTLV-1, HCV	23
**Total**				**535**

B-CLL, B-cell chronic lymphocytic leukemia; HIV-1, Human immunodeficiency virus 1; CMV, Cytomegalovirus; HTLV-1, Human T-lymphotropic virus 1; HCV, Hepatitis C.

### 3.1 Target cells

*What cells/tissues have been studied?* In terms of target cells, most studies (18/29; **Table 2**) have focused on lymphocyte kinetics, partly because subset kinetics are critical to the formation and maintenance of immune memory (e.g. #7,17,22,25) but also because lymphocyte kinetics may be pivotal to pathology as in HIV infection or lymphocytic leukemia (see below). Neutrophils have been the target in 2/29 studies cited (#9 and 18 – the latter used both deuterated glucose and water but here we refer only to deuterated water data). Adipocyte turnover has been the target in 9/29 studies cited here (**Table 2**). The third main application has been the study of skin turnover and metabolism, also allowing measurement of keratin kinetics without skin biopsy (19).

**Table 2:** Methods and applications of deuterated water cell turnover studies.

ID	Product (%)	Protocol		Prime duration (days)	*Equivalent Day1 Prime (ml)	Maximum Duration (weeks)	Target cells	†Adverse Events	‡ Dizziness Vertigo	Supplier	Modelling: 2H parameters	Normalization cell type
		Prime	Maintenance									
**1**	70 or 99	50ml every 3h	60-70ml	1	280	9	Lymphocyte	No	1	(A)	Urine and Saliva	Monocytes and granulocytes
**2**	70	70ml Seven times	70ml	1	343	9	Lymphocyte	NS	0	NS	Urine and Saliva	Monocytes or granulocytes
**3**	70	70ml ever 3-4 h then 50ml three times for 5 days	35-50ml twice	6	392	9	Adipose Tissues	No	1	(B)	Plasma and Urine	Monocytes
**4**	70	90ml twice	60ml	5	126	12	Lymphocyte	No	3	(A)	Plasma	No
**5**	70	50–150 ml for 1–4 weeks		NS	NS	4	Epithelial cell	No	0	NS	Urine and Saliva	Monocytes
**6**	70	70ml three times	50ml twice for 23 days	5	147	4	Adipose Tissues	NS	0	NS	Saliva	NS
**7**	99^¶^	10ml per kg body water	1/8 of this initial dose	1	420	9	Lymphocyte	NS	0	(A)	Urine	Granulocytes
**8**	NS	90ml twice	60 ml	5	180	12	Lymphocyte	NS	0	NS	Plasma	NS
**9**	99^¶^	10ml per kg body water	1/8 of this initial dose	1	420	9	Neutrophils	NS	0	(A)	Urine	Granulocytes
**10**	70	50ml three times	60ml	5	105	6	Lymphocyte	NS	0	(C)	Saliva	No
**11**	NS	NS		NS	NS	12	Lymphocyte	NS	0	NS	Plasma or Saliva	No
**12**	70	50ml twice or three times for 14 to 28 days		NS	NS	4	Epithelial cell	No	0	(C)	Saliva and Serum	Monocytes
**13**	70	480ml	60 or 80ml	1	336	9	Lymphocyte	No	0	(A)	Plasma	Granulocytes
**14**	99	7.5 ml per kg body water	1.25ml per kg body water	1	315	9	Lymphocyte	NS	0	(A)	Urine	Granulocytes
**15**	70^¶^	70ml ever 3-4h^¶^ then 40 mL three times for 5 days	35-50ml twice	6	392	4	Adipose Tissues	NS	0	NS	NS	NS
**16**	99	35mL three times	35mL twice	7	105	8	Adipose Tissues	NS	0	(D)	urine	Monocytes
**17**	70	50ml three times	50ml twice	7	105	7	Lymphocyte	NS	0	NS	Saliva	NS
**18**	70	50ml three times	50ml twice	7	105	7	Neutrophils	NS	0	(A)	Saliva and Urine	NS
**19**	70	50ml three times	50ml twice	5	105	8	Lymphocyte	NS	0	(A)	Plasma or Saliva	NS
**20**	70	50ml three times	60ml once	5	105	4	Lymphocyte	NS	0	(A)	Plasma	NS
**21**	99	35mL three times	35mL twice	7	105	8	Adipose Tissues	NS	0	(D)	Urine	NS
**22**	70	50ml three times	50ml twice	7	105	7	Lymphocyte	NS	0	NS	Saliva	NS
**23**	70	50ml three times	50ml twice	7	105	7	Lymphocyte	NS	0	(A)	NS	NS
**24**	70	140mL divided then 40mL three times for 5days	40mL twice	6	98	8	Adipose Tissues	No	Yes	(B)	Urine	NS
**25**	70	50ml three times	50ml twice	7	105	7	Lymphocyte	NS	0	NS	Saliva	Granulocytes
**26**	99	7.5ml per kg body water	1.25ml per kg body water	1	315	6	Lymphocyte	NS	0	(A)	Urine	Granulocytes
**27**	99^¶^	7.5ml per kg body water	1.25ml per kg body water	1	315	5	Lymphocyte	NS	0	NS	Urine	Granulocytes
**28**	99	35mL three times	35mL twice	7	105	8	Adipose Tissues	No	0	(D)	Urine	Monocytes
**29**	70	50ml three times	50ml twice	7	105	7	Lymphocyte	NS	0	NS	Saliva	Monocytes
**Mean**				**4.6**	**205**	**7.5**						

NS, Not stated. * Calculated for 70kg Man and expressed as volume of 99% ^2^H_2_O; †Adverse events exclude vertigo or dizziness which are known transitory effects of heavy water - no studies reported deuterium toxicity; ‡ Study ID #1 reported 1 case from 21 participants, #3 reported 1 from 19, #4 reported 3 from 19 and #24 reported a “transient lightheaded feeling at the beginning of the labelling period” but did not give numbers; ¶ not explicitly stated but assumed from other studies by the same group. Suppliers: (A) Cambridge Isotopes, (B) Isotec (Miamisburgh, OH), (C) Spectra Gases Inc, (D) Sigma-Aldrich.

### 3.2 Patient groups/disease states

*Who has it been used in?* Since our knowledge of normal homeostatic cell proliferation is so limited, many studies (10/29; #1,3,6,7,9,14,17,18,19,22; **Table 1**) have focused on defining parameters in normal healthy adult humans. Other studies (11/29; #2,13,15,16,21,23,24,25,27,28,29; **Table 1**) have compared people with conditions such as HIV/HCV/CMV infection, diabetes, or obesity with healthy volunteers. Clearly cell proliferation is a critical readout in cancer biology but application in this arena has been limited (8/29; #4,5,8,10,11,12,20,26; **Table 1**) and largely limited to studies in patients with leukemia (e.g. #4,8,10,11,20).

Deuterated water has not to date been used to study cell turnover in children or pregnant women, who are usually excluded in study protocols, although it has been used to study body composition in both settings – albeit at lower enrichments (43, 44). Patients with end-stage renal disease have not been studied although, interestingly, they have lower levels of body water ^2^H compared to people without renal disease suggesting that renal dysfunction selectively removes more ^2^H than ^1^H (45). The difference is on a scale of about 0.0005 atoms percent excess (APE) (7) so can be disregarded for cell proliferation studies.

### 3.3 Dose and duration used for previous studies

The way in which deuterated water is used varied by study protocols in terms of formulation, the use (or not) of a priming dose, the steady state dose/target plateau level, and the duration of dosing. The studies cited here have used either 70% deuterated water (19 studies) or 99.9% (9 studies); in two studies the formulation was not specified (**Table 2**). All gave the dose orally.

#### 3.3.1 Prime

**Figure 1:**
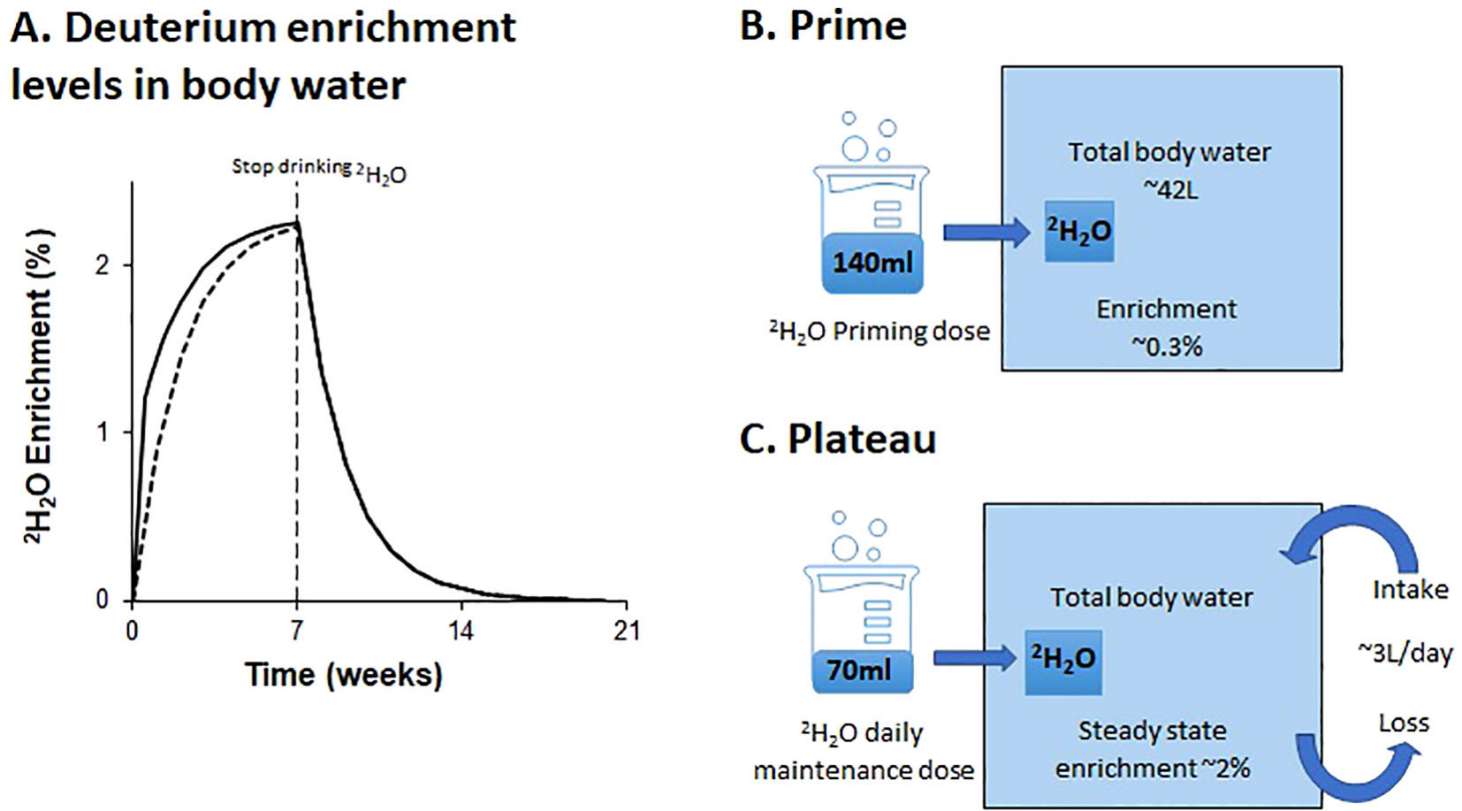
Schematic of predicted deuterium enrichments in body water with oral labeling. The schematic **(A)** shows deuterium enrichment levels in body water over 20 weeks during and after a 7-week labelling phase with a priming dose (solid line), and without a priming dose (dashed line); **(B, C)** illustrate the impact on deuterium enrichment of a typical dose of deuterated water (200 ml of 70% on day one, followed by 100 ml of 70% daily thereafter). Amounts are shown as 100% deuterated water equivalents.

In general, most study protocols (26/29) included administration of priming dose to more rapidly achieve the desired labelling rates (**Figure 1A**); without priming, enrichment will rise as an exponential to a plateau. The level achieved with a priming dose is determined by the ratio of the priming dose to total body water volume (6). Hence, since total body water is roughly 0.6 L/kg body weight for males and 0.5L/Kg for female (46), for a 70kg man to achieve a labelling enrichment of 1% would require a prime of ~420 mL deuterated water (or the equivalent, 600ml of 70% enriched water). Priming doses in this range were given in several studies (#7 and 9; **Table 2**) but most studies compromised and used a lower dose (as illustrated in **Figure 1B**), presumably intended to expedite the achievement of plateau whilst minimizing the likelihood of dizziness. Doses in the range of 140–200ml of pure deuterated water per day for 1-7 days were more typical.

#### 3.3.2 Maintenance dose

The plateau level is determined by the ratio of the rate of administration to the rate of water flux. The latter is largely a behavioral and environmental (rather than physiological) parameter and, as such, may be highly variable (47). We find participants typically achieve enrichments of ~2% deuterium in saliva when consuming doses equivalent to 70 mL of pure deuterated water daily; this equates to a water intake/loss rate of about 3L/day (**Figure 1C**). Most published studies appear to target body water deuterium enrichments of around 1.0-2.5%, although only a few explicitly reported their target range. The desired level will depend upon the rate of division of the target cell and the sensitivity of the downstream analytic pathway. The estimated maximum maintenance dose of deuterated water for a 70 kg male was 105 ml per day. Studies #5 and #12, which labeled epithelial cells, utilized a higher maintenance dose compared to other studies aiming to achieve similar deuterium enrichments to other studies without the need for a priming dose (**Table 2**).

#### 3.3.3 Duration

Since deuterated water labelling is generally chosen to analyze slow turnover cells, lengthy labelling periods predominate, ranging from 4 to 12 weeks (mean 7.5 weeks) (**Table 2**). Lymphocyte studies tend to have longer labelling periods (8.1 weeks) than non-lymphocyte studies (6.6 weeks).

### 3.4 Modelling

All studies used some form of normalization, which is an essential step in data analysis to account for variations in the precursor (body water) deuterium enrichment, although not all publications explicitly stated the approach that had been taken. In addition to measuring the body water (precursor) deuterium enrichment, many studies analyzed a rapid-turnover cell population for DNA deuterium enrichment (**Table 2**), typically monocytes or granulocytes whose circulating cell populations will have been replaced several times during the labelling period. They yield a value for the maximum level of deuterium incorporation in DNA against which the enrichments in the cells of interest can be scaled to calculate fractional replacement rates (14). In this review, fifteen studies reported the use of either monocytes or granulocytes for normalization. DNA enrichment levels about 3.5-5.2 times higher than the corresponding body water enrichment were generally reported, as expected (14, 20, 48), reflecting the effective number of labeling sites. Saliva, urine, or blood samples were also collected to measure enrichment of deuterium in body water in 27/29 studies; most used saliva or urine as collection is less invasive and simpler than blood (49).

### 3.5 Toxicity

No specific reports of toxicity or adverse effects were reported in any of the studies cited. Nine studies specifically stated the absence of adverse events; one study (#28) reported a potential adverse event which was related neither to the trial nor to deuterated water administration; the remaining studies did not mention adverse effects. There may have been some under-reporting of expected effects such as vertigo, light-headedness or dizziness as these are known and expected transient effects of changes in water density (12). We found four reports which did mention dizziness. Study #1 reported 1 case of “transient dizziness … resolved within 30 minutes” from 21 participants; #3 reported 1 case from 19 where “… a mild, transient light-headed feeling was described by one subject”; #4 reported 3 cases from 19 where “a transient sense of light headedness during the loading phase”; #24 reported a “transient lightheaded feeling at the beginning of the labelling period” but did not give numbers of participants affected. Two of these studies (#1 and #3) used a significant priming dose on the first day (estimated 280 and 392ml respectively), confirming the supposition that such effects are more likely with higher doses. Conversely, studies #4 and #24, which reported side-effects, used relatively small priming doses on the first day (estimated 98 and 126ml respectively), whilst several studies which used higher priming doses (Study ID #7,9,13,14,15,26,27) did not report dizziness or other adverse events at all.

## 4 Discussion

This review documents the current status of human *in vivo* labelling studies using deuterated water to investigate cell turnover. The most striking observation is how extensive international experience is. We identified 535 participants in 29 such studies to date, representing about 520 individual subjects, including both healthy volunteers and participants with specific clinical conditions. Most studies focus on lymphocyte kinetics because of the fundamental link between cell kinetics and the generation and maintenance of immune memory, but perhaps also because the initial driver to the development of these *in vivo* labelling techniques was an urgent need to understand lymphocyte depletion in HIV infection (3). Clearly there is great scope for further application to other cell-types and in other settings with adaptation of the protocol to suit both the cell of interest and the clinical scenario.

In terms of protocols and practice, it is not appropriate to make uniform recommendations for labelling rate and duration as the best option is determined by the target cell and the sensitivity of the analytic instrumentation; hence there is no one “right” protocol to follow. A rapidly-dividing cell may reach readily measurable DNA labelling rates after only a short period (18, 33), whereas more slowly-dividing cells will require longer and/or higher rates of precursor enrichment (26) to achieve measurable deuterium enrichments. In this review we noted a range of durations from 4 to 12 weeks and a range of doses up to the equivalent of 100ml per day of pure heavy water (28, 30). Very rapidly-dividing cells such as granulocytes, monocytes, and dendritic cells are probably better traced with deuterated-glucose which has a very small pool size and high turnover rate resulting in rapid ‘on’ and ‘off’ precursor labelling, although the two approaches may yield different parameter estimates (48). Glucose labelling studies were considered beyond the remit of this review and are not discussed further.

**Figure 2:**
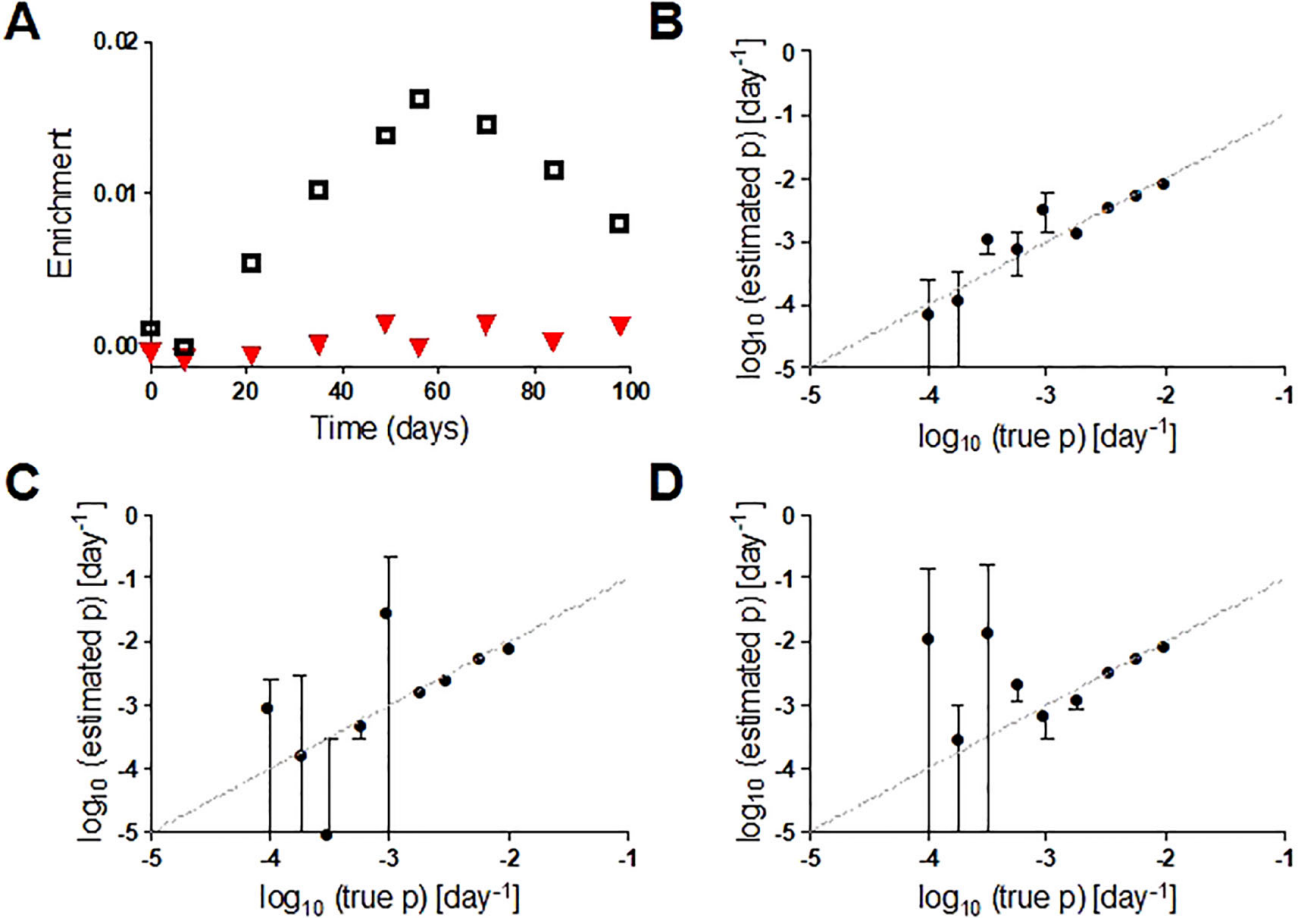
**(A)** Representative in silico data for deuterium enrichment in DNA in two cell populations with different proliferation rates (p, ▼ p=0.0002 d^-1^; □ p=0.006 d^-1^). **(B)** Comparison of true value of p (x axis) with estimated value (y axis) for a 7-week labeling protocol with 7-weeks post-administration follow-up and 9 sampling points. Bars represent standard errors (asymptotic covariance matrix method) and dashed line is line of equality. **(C)** As for B but with 6 rather than 9 data points; **(D)** as for B but with 4 weeks rather than 7 weeks of labelling. See **Supplementary Material** for details.

If designing a new study with deuterated water, investigators should consider either an *in silico* model or a pilot study or to determine the optimum dose and duration needed to detect the anticipated turnover rate, especially when the turnover rate of target cells is uncertain (50). The protocol should target achievement of a cell enrichment within the optimal analytic range for isotope enrichment analysis in the local mass spectrometry facility – a worked example is shown in **Supplementary Material**. Pragmatic concerns may need to be balanced against the theoretical ideal. For example, more sampling points will increase confidence in estimated turnover rates but make the study more onerous and less acceptable to research participants and regulatory bodies. The impact of variations in the number of sampling points and duration of labelling on parameter estimates can be simulated. **Figures 2A–D** shows how the reliability of the estimate of cellular proliferation rate depends on the proliferation rate itself, the number of sampling points and the duration of labelling. So, for example, if seeking to measure the proliferation rate (p) of a cell where it is anticipated to be <10^-3^ day^-1^, reducing the labeling time from 7 to 4 weeks substantially increases the error of the parameter estimate for p (**Figures 2B, D**).

Protocol adherence is clearly critical; this can be monitored practically in real-time through the return of empty deuterated water bottles and also, later, analytically by monitoring of deuterium levels in body water (urine, saliva, plasma) or rapidly-labeled cell samples (monocytes, granulocytes). Protocols may also be optimized to minimize consumption of deuterated water on economic grounds; prices have risen dramatically, over 5-fold in a decade. Of the 16 studies which mentioned their source, all used products from one of four US companies, some of which shared suppliers.

Modelling is essential to extract meaningful biological parameters from labelling data. The impact of model choice on parameter estimates has been discussed elsewhere (51–53) so we do not review it further here. Some studies estimated cell kinetics from only the up-labeling phase or only the de-labeling phase (32). With such study designs, only the net accumulation of label can be quantified (the resultant of cell proliferation and cell loss); it is not possible to separate proliferation and loss. Modelling needs to account for cells entering and leaving a subset or compartment by phenotype change as well as by proliferation and cell death (35, 54). Furthermore, where T cell labelling is measured in blood, consideration must be given to the fact that, at any one time, most lymphoid cells are not in blood but in lymphoid organs and other anatomical compartments. The blood compartment represents a conduit for trafficking cells which egress to tissues and recirculate. Although direct sampling of human tissues may not be possible for logistic and pragmatic reasons, it may be possible to draw inferences about how cells traffic between compartments from blood labelling and other data (42, 55).

Collection of cells for normalization was mentioned in 15 studies. Without normalization estimates must be based on use of a constant correction factor (variously referred to as c or b_w_ in the literature). However given that, for reasons that remain unclear, this factor can vary considerably between individuals (e.g. in *Zhang* et al. (42), b_w_ varied from a minimum of 3.9 to a maximum of 6.2 with a mean of 5.0), and given that errors in b_w_ directly propagate into errors in estimated proliferation we suggest that future studies should therefore use personalized normalization estimates based on a fully-labelled cell such as a granulocyte or monocyte in their protocol design.

No clinical toxicity signals were noted in any of the studies we identified which included experience in 535 participants. Some reports of transient dizziness were documented, as expected and potential participants should be warned of this possibility. The long safety record of deuterium as a tracer for human studies (10, 11) therefore seems to be borne out in these cell turnover tracer studies. This is significant as cell turnover studies tend to target tracer levels in the 1-2% enrichment range, slightly higher than previous body composition assessments. There are, of course, many other reports of human studies using deuterated water as a tracer over the same period of time which did not target cell turnover as a read-out; such studies have not been included in this review.

This is not to say that stable isotopes never have toxicities. At very high levels of deuterium water enrichment biochemical and physiological effects are seen. Although deuterium (^2^H) is chemically identical to protium (^1^H), with the same electronic structure and the same number of protons, the presence of the extra neutron changes the energy of the bonds deuterium forms (versus protium); this can cause the rate of chemical reactions to change (56). Physiologically one effect of this is interference with mitotic spindle formation resulting in reduced rates of cell proliferation (57). Indeed, on this basis, it has been suggested that deuterium depletion, below naturally-occurring levels, might suppress tumor growth and increase apoptosis (58, 59). Conversely, at not dissimilar levels (25-30%), deuterated water impaired tumor cell growth in in a mouse human pancreatic tumor model (60) and in non-small cell lung cancer cell lines (through microtubule depolymerization and inhibition of PI3K/Akt/mTOR signaling) (61).

Dose and duration of exposure are clearly critical (10, 11). All the animal studies showing clinical impact were performed at very high levels of enrichment. For example, experiments showing reproductive impairment in rodents used 25% deuterium-enriched drinking water (62); those in two dogs documenting electrolyte imbalance, neuromuscular dysfunction and progressive lymphopenia and agranulocytosis targeted 20/35% labelling (63).

*In vivo* human deuterated water studies never approach these levels and are based on a long track record of the safe use of deuterated water for other purposes prior to its use to measure cell proliferation in applications. These include body water estimation, body composition studies, and, in combination with oxygen-18, the measurement of free-living energy expenditure in humans (64, 65). Such studies, using deuterated water at tracer doses, have not been associated with significant toxicity (10, 11). Cell turnover studies, whilst not systematically searching for sub-clinical toxicities, add to this body of evidence supporting the safety of deuterium as a tracer in human studies.

This scoping review documents the extensive current experience with the deuterated water methodology as applied to human cell kinetics but the implications go beyond cell turnover studies. Deuterium use in human studies is likely to increase with the ongoing development of novel metabolic imaging modalities (66, 67). The published studies reviewed here give a strong signal about safety and the absence of clinical toxicity. The extensive diversity of protocols probably reflects the diversity of applications for this approach as well as local, pragmatic and practical considerations but some lessons are clear – these include the need for a targeted approach generating enrichments in the analytic range, the value of *a priori* in silico simulation, appropriate dose and duration of label, sufficient sampling points, normalization against a fully-labeled cell, and apposite modelling. The published literature cited here provide important baseline information for the development of new applications of this powerful approach and reassurance for potential participants and regulatory authorities.
