# Supplementary material for: Deuterated water (2H2O, heavy water) labelling to investigate human cell dynamics in vivo - lessons in protocol design and toxicity from the current literature
Source: Front Immunol. 2025 Apr 28;16:1544193. doi: 10.3389/fimmu.2025.1544193 (PMC12066605; doi:10.3389/fimmu.2025.1544193)
Supplement: Supplementary file 1 [file DataSheet1.pdf]

Supplementary material

1. Rough worked example - Predicting enrichment of labelled cells to align with analytic capabilities

A rough worked example can be made to estimate impact of dosing on measured deuterium enrichments of DNA in labelled cells.

Input parameters:

a. Estimated proliferation rate of cells of interest
b. Rate of administration of deuterated water
c. The analytic range and precision of the mass spectrometer used

Assumptions

a. Body water volume ~42L for adult male – adjust for other categories of participant
b. Body water turnover ~3L/day – a behavioural variable
c. The number of labelled deuterium atoms per analysed moiety, or 'scaling fator' (bw or c) – see Discussion

Suppose:

(i) we wish to study a cell whose proliferation rate is expected to be about 1% per day;
(ii) we give deuterated water as shown in **Figure 1** at a rate of 200 ml of 70% on day one, followed by 100 ml of 70% daily thereafter.

In this case, we will achieve deuterium enrichments in body water of about 1-2% APE over the course of the labelling phase – although modelled dynamically let us take a constant value of 1.5% for the sake of this approximate illustration.

For a cell proliferating at about 1%/day, after 1 week ~7% of cells will be 'new'.

Each new cell will be labelled according to {the proportion of deuterium-enrichment in all hydrogens (~1.5% or x ~0.015)} x {the number of labelled deuterium atoms per analysed moiety (~5)}, so the expected enrichments in DNA will be:

The proportion of 'new' cells, 0.07 x 0.015 x 5 = 0.005 or 0.5% APE

(iii) we have a mass spectrometer which measures deuterium enrichment with a typical standard deviation of replicates of 0.05 atoms percent enrichment (APE)

On analysis the expected value for deuterium enrichment then represents a z-score of 10 (0.5 / 0.05) so should be readily measurable with a high degree of accuracy.

At 7 weeks, because some new cells are lost, the proportion of new cells will be ~39%, so predicted labelling is 0.39 x 0.015 x 5 = 2.9%, well in the measurement range.

2. In silico predictions of enrichment of labelled cells

More accurate predictions can be made *in silico* by modelling that takes into account the dynamics of deuterated water and cell labelling. This would be achieved in the following manner:

Construct a model to describe label in your cell population of interest or use an existing model (equation 3 in [1] is a generic model suitable for many cell populations). Reproduced here for ease of reference:

$$\dot{L} = pb_w S(t) - d^* L$$

Where

$$S(t) = \begin{cases} f(1-e^{-\delta t}) & t \le \tau \\ f(1-e^{-\delta\tau})e^{-\delta(t-\tau)} & \tau < t \end{cases}$$

Substitute in realistic numerical values (for example for this model the parameters *bw*, *f* and *δ* are all tabulated in Supplementary Table 3 in [1], medians could be used). By definition the kinetic parameters of the cell population (*p* and *d**) are unlikely to be known but a range of plausible guesses can be made, in turn, spanning the range where the true value is thought to lie. For each of these values of *p* and *d**, substituting them into the equations and solving will give a predicted value of label over time for a given choice of labelling period τ. Points can then be "sampled" at times that reflect the sampling strategy of interest, noise added (a random variable from a normal distribution with mean zero), and added to the "sampled label". In this way an *in silico* data set (for known *p* and *d**) can be generated. From here, you follow the proposed modelling protocol as if this was your observed data, fitting the choice of model (not necessarily the generating model) to the *in silico* data and the parameters estimated, giving $\hat{p}$ and $\hat{d}$*. The discrepancy between the true (generating) values of *p* and *d** and their estimates $\hat{p}$ and $\hat{d}$* can be assessed. If the errors are too large then the experimental protocol will need to be altered – perhaps a different length of labelling period or a larger number of samples, until the level of error is considered acceptable. This process will then need to be repeated for all values of *p* and *d** that span the potential range.

1. Zhang, Y. *et al.* KIR-HLA interactions extend human CD8+ T cell lifespan in vivo. *J. Clin. Invest.* **133** (2023).
